# Spatial distribution of *Glossina sp*. and *Trypanosoma sp*. in south-western Ethiopia

**DOI:** 10.1186/s13071-015-1041-9

**Published:** 2015-08-19

**Authors:** Reta Duguma, Senbeta Tasew, Abebe Olani, Delesa Damena, Dereje Alemu, Tesfaye Mulatu, Yoseph Alemayehu, Moti Yohannes, Merga Bekana, Antje Hoppenheit, Emmanuel Abatih, Tibebu Habtewold, Vincent Delespaux, Luc Duchateau

**Affiliations:** Department of Clinical studies, College of Veterinary Medicine and Agriculture, Addis Ababa University, P.O.Box 34, Bishoftu, Oromia Ethiopia; Department of Comparative Physiology and Biometrics, Faculty of Veterinary Sciences, Universiteit Gent, Salisburylaan 133, B-9820 Merelbeke, Belgium; National Tsetse and Trypanosome Investigation and Control Centre, P.O.Box 13, Illu-Aba-Bora, Bedelle Ethiopia; National Animal Health Diagnostic and Investigation Centre, P.O. Box 04, Sebeta, Oromia Ethiopia; International Maize & Wheat Improvement Centre (CIMMYT), Socio-economics Program, Global Cereal Rust Monitoring system, P.O. Box 5689, Addis Ababa, Ethiopia; Department of Microbiology and Veterinary Public Health, School of Veterinary Medicine, Jimma University, P.O. Box 307, Jimma, Ethiopia; Institute for Parasitology and Tropical Veterinary Medicine, Freie Universitaet Berlin, Robert-von-Ostertagstr. 7-13, 14163 Berlin, Germany; Department of Biomedical Sciences, Institute of Tropical Medicine, Nationalestraat 155, Antwerp, Belgium

**Keywords:** Trypanosoma, Glossina, Ethiopia, Cattle, Risk factors

## Abstract

**Background:**

Accurate information on the distribution of the tsetse fly is of paramount importance to better control animal trypanosomosis. Entomological and parasitological surveys were conducted in the tsetse belt of south-western Ethiopia to describe the prevalence of trypanosomosis (PoT), the abundance of tsetse flies (AT) and to evaluate the association with potential risk factors.

**Methods:**

The study was conducted between 2009 and 2012. The parasitological survey data were analysed by a random effects logistic regression model, whereas the entomological survey data were analysed by a Poisson regression model. The percentage of animals with trypanosomosis was regressed on the tsetse fly count using a random effects logistic regression model.

**Results:**

The following six risk factors were evaluated for PoT (i) altitude: significant and inverse correlation with trypanosomosis, (ii) annual variation of PoT: no significant difference between years, (iii) regional state: compared to Benishangul-Gumuz (18.0 %), the three remaining regional states showed significantly lower PoT, (iv) river system: the PoT differed significantly between the river systems, (iv) sex: male animals (11.0 %) were more affected than females (9.0 %), and finally (vi) age at sampling: no difference between the considered classes. Observed trypanosome species were *T. congolense* (76.0 %), *T. vivax (18.1 %), T. b. brucei (3.6 %),* and mixed *T. congolense*/*vivax* (2.4 %).

The first four risk factors listed above were also evaluated for AT, and all have a significant effect on AT. In the multivariable model only altitude was retained with AT decreasing with increasing altitude. Four different *Glossina* species were identified i.e. *G. tachinoides* (52.0 %), *G. pallidipes* (26.0 %), *G.morsitans submorsitans* (15.0 %) and *G. fuscipes fuscipes* (7.0 %). Significant differences in catches/trap/day between districts were observed for each species. No association could be found between the tsetse fly counts and trypanosomosis prevalence.

**Conclusions:**

Trypanosomosis remains a constraint to livestock production in south-western Ethiopia. Four *Glossina* and three *Trypanosoma* species were observed. Altitude had a significant impact on AT and PoT. PoT is not associated with AT, which could be explained by the importance of mechanical transmission. This needs to be investigated further as it might jeopardize control strategies that target the tsetse fly population.

## Background

In sub-Saharan Africa, trypanosomosis is responsible for poverty, weak economic growth and low agricultural production resulting in subsistence livelihood [1–4]. In regions under challenge of trypanosomosis, land cannot be exploited for livestock rearing. The resulting lack of draught power is further compromising crop production. In rural Africa, livestock breeding constitutes an alternative banking system and contributes to social wealth and welfare. However, 48 million (=30.0 %) African cattle, not including other livestock species, are exposed to trypanosomosis [5].

Ethiopia covers an area of 1.1million km^2^ with 240,000 km^2^ of fertile areas under threat of trypanosomosis. Particularly affected are the western and southern lowlands, preventing agricultural activities. Drastic droughts in the 70’s and early 80’s have caused a significant number of people to move from the northern highlands to the tsetse-infested south-western region in search of fertile land [6]. Since the 70’s, this situation has worsened by repeated abnormal climatic fluctuations linked to El Niño [7]. Furthermore, new governmental land use regulations that were adopted between 1987 and 2005 resulted in a threefold increase in utilised agricultural land [8, 9]. This “demographic” clearing, whose effects are comparable to the bush clearing strategy of colonial Africa, substantially changed the distribution and abundance of the different tsetse species, deemed to be the most important vector for trypanosomes [10]. Currently, 14.8 million cattle, 6.1 million sheep and goats, and 1.2 million equines are at risk of trypanosomosis in this recently settled south-western region. Tsetse control is organised by the Ethiopian government through NICETT (National Institute for the Control and Eradication of Tsetse and Trypanosomosis); formerly represented by STEP (Southern Tsetse Eradication Program). Their strategies comprise the use of insecticidal pour-ons and insecticide impregnated traps and targets. Complementary to those vector control activities, trypanocidal drug treatment remains the most widely used control strategy because it is available and most affordable for livestock breeders. Trypanocides minimize the impact of the parasite on animal health and also reduce the period that the animal is infectious for possible vectors [11]. The presence of single and multiple drug resistant trypanosome strains in different locations [12–14] is hampering the success of chemotherapeutic and prophylactic approaches. For decades, the association of vector control and chemotherapy has been used to minimize the risk and impact of trypanosomosis [15]. However, despite more than 30 years of various attempts of tsetse and trypanosomosis control, tsetse flies have expanded their distribution and reinvaded previously tsetse-free areas at a rate of 200 m/year [16, 17]. This failure in achieving sustainable results is explained through fragmented and uncoordinated actions induced by poor information coupled with the absence of long-term coherent policies [18]. Tsetse populations are highly resilient; populations seem to restore and expand as soon as control measures are discontinued [19]. Accurate data on vector and parasite distributions as well as on risk factors for trypanosomosis among the domestic hosts are of paramount importance to control the disease with the ultimate goal of achieving eradication.

Despite the importance of such data, the last country-wide census on tsetse flies and trypanosomosis was conducted by Langridge in 1976 [20]. The aim of this study was thus (i) to update the available data by conducting large entomological and parasitological surveys and (ii) to identify the main risk factors influencing the transmission of the disease.

## Methods

### Study region

**Fig. 1:**
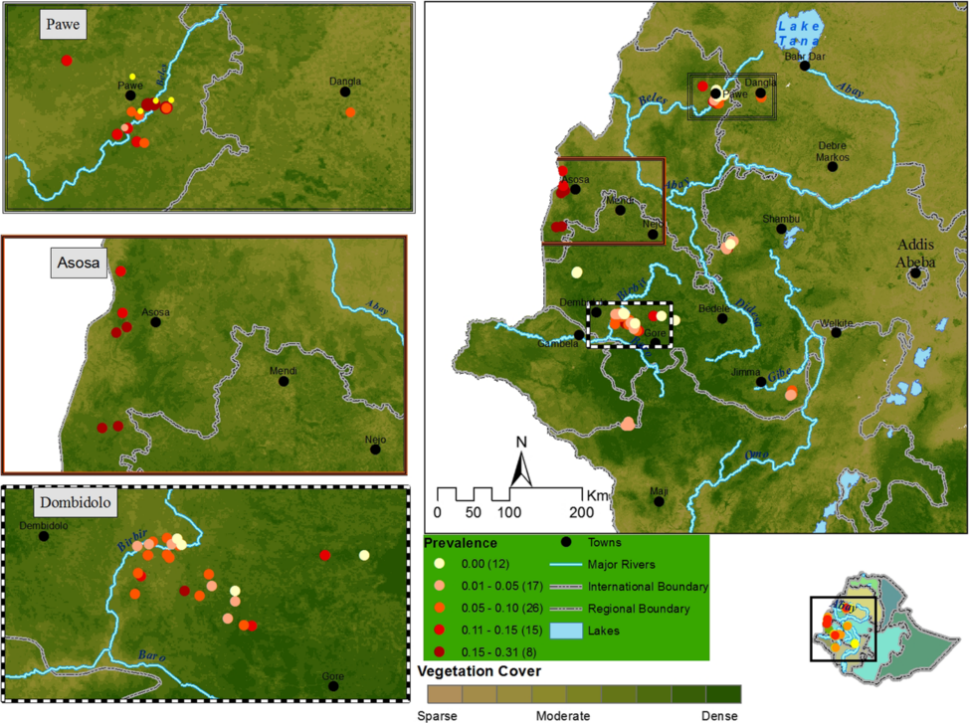
Map of the study area showing the selected Peasant Associations, trypanosome prevalence and distribution

**Fig. 2:**
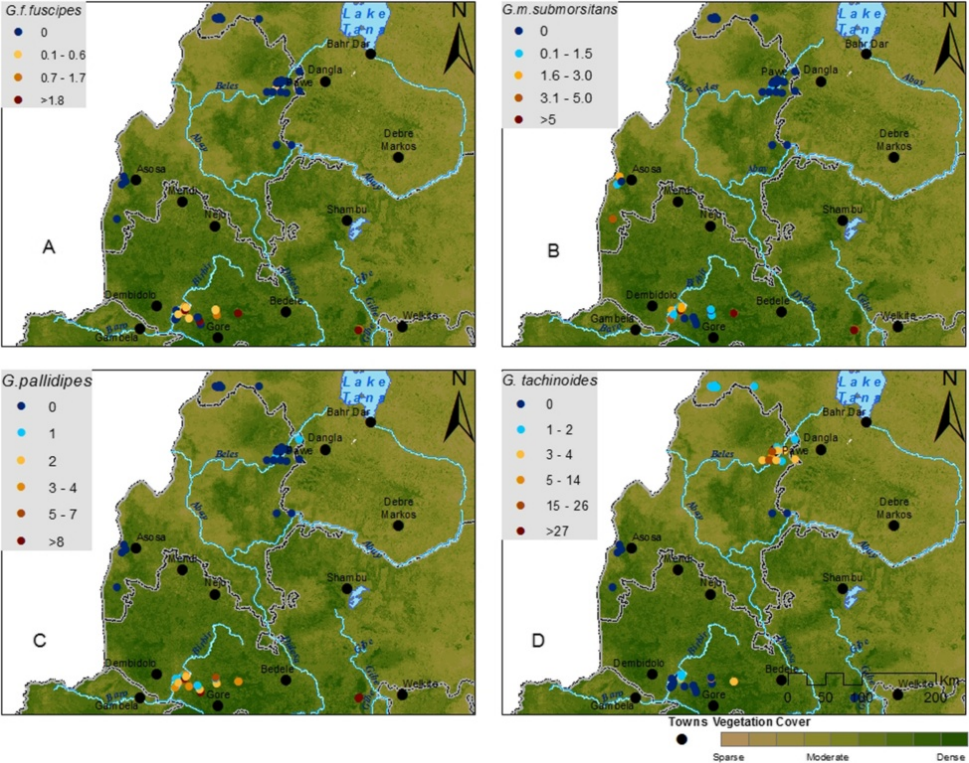
Map showing the selected Peasant Associations: tsetse fly catches and distribution

The study was conducted in south-western Ethiopia. The parasitological survey was conducted in a region located between 7°12′ to 10°09′N latitude and 34°39′ to 35°00’E longitude and the entomological survey between 8°22′ to 12°13′N latitude and 35°32′ to 37°28′E longitude (Figs. 1 and 2). The altitudes of the study regions ranged between 1040 and 2012 m and between 628 and 1673 m.a.s.l. (metres above sea level) for the parasitological and the entomological surveys, respectively. The region contains a hydrographic network including the Abay (Blue Nile), Didessa, Anger, Baro Akobo, Birbir, Dabus, Ghibe and Tekeze rivers and their tributaries. The Abay Didessa has a runoff of 52.6 billion m^3^/year and covers 199,812 km^2^ catchment area with elevation ranging from 500 to 4261 m. The Boro-Akobo has 23.6 billion m^3^/year runoff and covers 75,912 km^2^ catchment area with elevation ranging from 390 to 3244 m. The Ghibe-Omo river has 17.9 billion m3/year runoff, 79,000 km2 catchment area and a variety of wildlife parks [21]. There are two rainy seasons: the short one from the end of February to the end of April and the long one from June to September. The current study area includes four of the ten ecosystems of the country i.e. (i) woodland, (ii) lowland tropical forest, (iii) montane moist forest and (iv) montane dry evergreen [22]. The primary forests in these ecosystems together with the river networks constitute sanctuaries for tsetse flies and diverse wildlife [23] despite an intense deforestation rate. Firewood and land to feed the growing population are the main causes of encroachment [24]. Livestock is mainly fed on crop residues (at the end of the rainy season, beginning of the dry season) and on naturally persistent pastures. As most of the land is intensively cultivated during the long rainy season, cattle are moved to the vicinity of the forests where permanent grassland is available to avoid damage to the crops. Cattle are the dominant livestock species. The Horro breed is concentrated in the southwest and west of Ethiopia, the Abigar (Nuer) breed in the Gambela region, the Gurage breed in the Gurage zone and the Fogera breed in northwest Ethiopia in Amhara region [25].

Trypanosomosis occurs in five of the nine Ethiopian regional states (Oromia, Amhara, Gambela, Benishangul-Gumuz and Southern Nations Nationalities and Peoples Region) comprising 240,000 km^2^ of land area. Sampling was only carried out in 4 states (excluding Southern Nations Nationalities and Peoples Region). From the 4 regional states, eleven districts were purposively selected based on the burden of trypanosomosis reported by farmers and based on the expert opinion of local veterinarians. Local experts used financial cost, morbidity, mortality and withdrawal from draft power as criteria to estimate the trypanosomosis burden. The 11 districts comprise a total of 340 peasant associations (PA). A PA is the lowest administrative structure in Ethiopia with an average land area of 53 km^2^. The tributaries in the vicinity of each PA (within a 3.5 km radius) were allocated to one of the four main river systems, i.e., Ghibe, Baro-Akobo, Abay (Blue Nile) and Abay-Didessa.

### Sampling frame

Two-hundred PAs served as sampling frame since 140 had to be excluded from the study due to poor accessibility.

A total of 30 PAs (Fig. 1) were selected at random from the accessible 200 PAs, and both a parasitological and entomological survey was carried out in those 30 PAs. In another set of 52 randomly selected PAs, only a parasitological survey was done, and in another set of eight randomly selected PAs, only an entomological survey was carried out. Therefore, an entomological survey was conducted in 38 PAs in seven of the above selected 11 districts. Additionally, only an entomological survey was done in 14 PAs that were randomly selected from 92 PAs in 3 districts (which were not a subset of the above 11 districts); thus entomological surveys were done in a total of 52 PAs in 10 districts in this study. These 3 districts (Quara, Chora Botor and Darimu) were purposively included. Quara district in north Amhara region, Chora Botor district in Oromia bordering Ghibe valley and Shewa highlands and Darimu district in Oromia bordering Birbir valley and Ilu-Abaabora highlands that have been reported to be the limit of tsetse fly distribution as per the information from Zonal Veterinary offices.

Within each PA selected for parasitological survey, a minimum of 75 heads of cattle were selected at random and sampled, which led to a total of 7021 animals from those 82 PAs in 11 districts (on average 85 heads of cattle/PA).

### Parasitological survey

Between 2009 and 2012, blood samples were collected from these 7021 animals. The geographical reference of the PA, the number of animals sampled per PA and their age (based on teeth method), sex and trypanosome infection status were recorded. Sampling took place during the 5 months of the dry season (October to February) and the 3 months of the short rainy season (March to May), but each PA was only sampled once. Blood was taken from the ear vein, collected in a heparinized capillary tube and centrifuged by the Micro-Haematocrit Centrifugation Technique [26]. A minimum of 50 fields were examined using a standard light microscopy at 400x magnification. Trypanosomes were identified according to their movement pattern and later confirmed by the examination of Giemsa stained thin smears [27].

The experimental protocol and sampling were approved by the Ethical Committee of Addis Ababa University and the Zonal Agricultural Bureau. The background of the study was explained to the Peasant Associations and consent of the farmers was asked for sampling of their animals.

### Entomological survey

For the entomological survey, a total of 1046 mono-pyramidal traps [28] were deployed in the 52 PAs. Three tsetse fly attractants (acetone, octenol and cow urine) were used [29, 30]. Traps were deployed for 2 days and flies were collected twice a day at around 10:00 AM and 6:00 PM. Tsetse flies were counted, species and sex were determined. The number of tsetse flies captured per trap per day was used as indicator of the AT.

### Data analysis

The relationship between the presence of trypanosomes and the potential risk factors of altitude, sampling year, regional state, river system, sex and age was modelled by a random effects logistic regression model with PA incorporated as random effect [31]. First, each potential risk factor was included alone in the model (univariate analysis), next, potential risk factors with a p-value lower than 0.10 in the univariate analysis were incorporated jointly [32], together with their two-way interactions (multivariate analysis), and the forward selection procedure was applied to obtain the final model [33]. The odds ratio with its 95 % confidence interval was used as summary statistic.

The AT of tsetse flies in each PA was given by the number of flies captured in all deployed traps. The relationship between the AT in a PA and the potential risk factors of altitude, sampling year, regional state and river system was modelled by a Poisson regression model with the number of traps in the PA included as fixed offset term [34]. First, each potential risk factor was included alone in the model (univariate analysis), next potential risk factors with a p-value lower than 0.10 in the univariate analysis were incorporated jointly, together with their two-way interactions (multivariate analysis), and the forward selection procedure was applied to obtain the final model. The incidence rate ratio (IRR) with its 95 % confidence interval was used as summary statistic.

The distribution of the ATs over the four different species was compared between the districts using the Kruskall-Wallis test. The difference of the total number of tsetse flies of the two sexes within each trap was compared for each *Glossina* species separately using the Wilcoxon rank sum test stratified for trap.

Finally, the relationship between trypanosome presence and AD was investigated by a random effect logistic regression model with PA incorporated as random effect, the trypanosome status of the animal as response variable and the AD in the PA as risk factor.

## Results

### Trypanosomosis prevalence, distribution and risk factors

Out of 7021 examined cattle, 675 (9.61 %) were trypanosome-positive. The predominant species was *T. congolense* (76.0 %), followed by *T. vivax* (18.1 %), *T. b. brucei* (3.6 %), and mixed *T. congolense*/*vivax* infections (2.4 %). Trypanosomosis prevalence varied significantly between the PAs. In 14.6 % of the PAs (*n* = 12), no trypanosomes were detected. The results are summarized in Fig. 1. When compared to Benishangul-Gumuz (18.0 %), the 3 other regional states had a significantly lower trypanosomosis burden: Amhara (12.0 %), Oromia (6.0 %) and Gambela (5.0 %).

In the univariate model (Table 1), low altitude was significantly and inversely associated with trypanosomosis. Geographical regions with an altitude lower or equal to 1200 m showed the highest trypanosomosis prevalence (12.0 %). Annual variation of the trypanosomosis burden was not significant between years. The highest and lowest trypanosomosis prevalence was observed at the first year (11.0 %) and the last year (4.0 %), respectively. Male animals (11.0 %) were significantly more often affected than females (9 %, *p* = 0.01). The distribution of trypanosomosis also varied significantly between the different river systems: the prevalence was correlated with the size of the river system with 5.0 % around the Ghibe river (17.9 billion m^3^/year), 7.0 % around the Boro-Akobo (23.6 billion m^3^/year), 11.0 % around the Abay/Blue Nile and 15.0 % around the enlarged Abay Didessa (52.6 billion m^3^/year) [21]. The different age classes did not differ significantly with respect to trypanosomosis burden. The highest and lowest trypanosomosis prevalence was observed in cattle of age > 4 years (11.0 %) and ≤ 2 years (6 %), respectively. In the final multivariable model, only altitude and sex had a significant effect (Table 2) confirming the trend of the univariate analysis.

**Table 1:** Distribution of cattle trypanosomosis among potential risk factors and univariate analysis

Risk factor	Category	N (N+)	Prevalence	95 % CI	p-value
Altitude	≤1200 m	971 (118)	12.2 %	10.2, 14.4	0.0002
	>1200 m, ≤1300 m	518 (43)	8.3 %	6.1, 11.0	
	>1300 m, ≤1400 m	910 (65)	7.1 %	5.6, 9.0	
	>1400 m, ≤1500 m	2936 (334)	11.4 %	10.2, 12.6	
	>1500 m, ≤1600 m	1074 (86)	8.0 %	6.5, 9.8	
	>1600 m	612 (29)	4.7 %	3.2, 6.7	
Year	2009	3191 (345)	10.8 %	9.8, 11.9	0.2154
	2010	2619 (255)	9.7 %	8.6, 10.9	
	2011	1054 (68)	6.5 %	5.0, 8.1	
	2012	157 (7)	4.5 %	0.18, 9.0	
Regional state	Benshangul-Gumuz	1763(312)	17.7 %	15.9, 19.6	0.0002
	Amhara	552 (65)	11.8 %	9.2, 14.8	
	Oromia	4214 (271)	6.4 %	5.7, 7.2	
	Gambela	492 (27)	5.5 %	3.6, 7.9	
Sex	Male	3356 (355)	10.6 %	9.6, 11.7	0.0102
	Female	3665 (320)	8.7 %	7.8, 9.7	
River system	Abay Didessa	1688(256)	15.2 %	13.5, 17.0	0.0419
	Abay	1294 (145)	11.2 %	9.5, 13.1	
	Baro Akobo	3377 (240)	7.1 %	6.3, 8.0	
	Ghibe	662 (34)	5.1 %	3.6, 7.1	
Age (years)	>4	4096 (435)	10.6 %	9.7, 11.6	0.2211
	>2, ≤4	2321 (201)	87 %	7.5, 9.9	
	≤2	604 (39)	6.5 %	4.6, 8.7	

With *N* number sampled, *(N+)* number positive, *CI* Confidence Interval

**Table 2:** The final multivariable model presenting the risk factors associated with trypanosomosis in Ethiopia

Risk factor	Category	Odds ratio	95 % CI	p-value
Altitude	≤1200 m^a^	1	-	-
	>1200 m, ≤1300 m	0.27	(0.14, 0.51)	<0.001
	>1300 m, ≤1400 m	0.40	(0.20, 0.80)	0.009
	>1400 m, ≤1500 m	0.53	(0.32, 0.90)	0.019
	>1500 m, ≤1600 m	0.32	(0.15, 0.65)	0.002
	>1600 m	0.34	(0.15, 0.74)	0.007
Sex	Female^a^	1	-	-
	Male	1.25	(1.06, 1.49)	0.01

With *CI* Confidence Interval, ^a^reference

### Distribution, abundance and risk factors of Tsetse flies

A total of 14,698 tsetse flies were trapped. Four different species were identified i.e. *G. tachinoides* (52 %), *G. pallidipes* (26 %), *G. morsitans submorsitans* (15 %) and *G. fuscipes fuscipes* (7 %). Significant differences in AD between districts were observed globally and for each of the *Glossina* species separately (*p* < 0.0001). The mean figure for AD was 7.45 catches/trap/day but considerable differences between PAs were observed. No catches were recorded at four PAs, i.e. Awjeben (Qwara district), Silga 22 (Asosa district), Tsiwuli and Yimale (Guangua district) and up to 78 catches/trap/day in the Pawe district. The AD and distribution across sampled PAs is shown in Fig. 2. *G. tachinoides* was the sole species trapped in the PAs of Guangua, Jawi, Pawe and Qwara districts. In the PAs of Asosa district, only *G. m. submorsitans* was trapped whilst all four tsetse species were detected in the PAs of Dalesadi and Dalewabara. Two species were observed in the PAs of Darimu district (*pallidipes* and *fuscipes*), of Hawagelan district (*pallidipes* and *submorsitans*), whereas in Choraboter district three species (*submorsitans*, *pallidipes* and *fuscipes*) were observed. More female than male tsetse were trapped with 53 % and 47 % respectively (*p* = 0.01). This was also the case for each species considered individually (Table 3).

**Table 3:** Distribution of tsetse flies by species, sex and district

District	n.s.	*G. m. submorsitans*			*G. pallidipes*			*G. f. fuscipes*			*G. tachinoides*			
		M	F	∑	M	F	∑	M	F	∑	M	F	∑	∑∑
Dalesadi	4	230	456	686	337	700	1037	78	151	229	74	74	148	2100
Dalewabera	4	197	320	517	198	388	586	147	220	367	163	207	370	1840
Choraboter	3	389	281	670	777	329	1106	207	150	357	-	-	-	2133
Hawagelan	3	69	121	190	127	247	374	1	6	7	-	-	-	571
Darimu	2	-	-	-	185	483	668	49	62	111	-	-	-	779
Asosa	1	41	93	134	-	-	-	-	-	-	-	-	-	134
Guangua	1	-	-	-	-	-	-	-	-	-	226	210	436	436
Jawi	1	-	-	-	-	-	-	-	-	-	341	350	691	691
Pawe	1	-	-	-	-	-	-	-	-	-	3081	2762	5843	5843
Qwara	1	-	-	-	-	-	-	-	-	-	62	109	171	171
∑		926	1271	2197	1624	2147	3771	482	589	1071	3947	3712	7659	14698
p-value		*p* < 0.001			*p* < 0.001			*p* = 0.047			*p* = 0.577			

With *n.s* number of species, *M* Male, *F* Female, *∑* = Total, ∑∑ = Grand total, p-values refer to comparison between sexes

**Table 4:** Average tsetse fly counts among potential risk factors and univariate analysis

Risk factor	Category	Average count	95 % CI	p-value
Altitude	≤1200 m^a^	17.1	16.6,17.6	<0.001
	>1200 m, ≤1300 m	12.0	11.6,12.5	
	>1300 m, ≤1400 m	13.6	13.2,14.0	
	>1400 m, ≤1500 m	5.9	5.5,6.2	
	>1500 m	12.0	10.9,13.2	
Year	2009	10.4	10.1,10.7	<0.001
	2010	18.3	17.9,18.7	
	2011	6.0	5.7,6.4	
Regional state	Benshangul-Gumuz	32.7	31.8,33.6	<0.001
	Amhara	3.3	3.1,3.5	
	Oromia	14.5	14.1,14.8	
River system	Abay Didessa	2.9	2.5,3.5	<0.001
	Abay	12.3	12.0,12.7	
	Baro Akobo	14.5	14.1,14.8	

In the univariate model (Table 4), all risk factors had a significant effect on AD, but only altitude was retained in the multivariable analysis. Low altitude was significantly and positively associated with AT. Geographical regions with an altitude lower or equal to 1200 m showed the highest average count of 17.1 catches/trap/day. Astonishingly, geographical regions above 1500 m showed an unexpected high average count of 12. This is linked to one single PA (Abuna Gali) where the AT was abnormally high.

Finally, no relationship between AT and trypanosomosis prevalence was found (OR = 1, 95 % CI: [0.99;1], *p* = 0.348).

## Discussion

### Trypanosomosis

The overall prevalence of trypanosomosis was 9.6 %. Using the same parasitological technique for diagnosis, a comparable prevalence was reported in north-western Ethiopia at Jawi district [35] and the western Oromia regional state [36]. Prevalence in the PAs ranged from 1.1 % to 30.8 %. *T. congolense* was the most predominant species (76.0 %) followed by *T. vivax* (18.1 %) and *T. brucei* (3.6 %). This is in agreement with the observations made by other groups in different tsetse-infested areas in Ethiopia [12, 20, 35–37].

Altitude is a well-known limiting factor for cattle trypanosomosis. The altitudinal difference in trypanosomosis burden is most often explained by the significant variation in tsetse densities across altitudes [38], but this point will be discussed later when describing the relationship between trypanosomosis and AT.

The annual variation in trypanosomosis prevalence was not significant from one year to another. However, the trend goes towards a decrease in prevalence, which is in line with the growing intensity of human encroachment and the resulting land use changes. It might also be explained by the intensity of control by NICETT but we do not have data to perform the analysis to substantiate it.

The highest prevalence of trypanosomosis was observed in PAs of the Asosa district where one single species of *Glossina* was trapped (*G. morsitans submorsitans*). This can be explained by the higher susceptibility of the morsitans group to infections by *T. congolense* [39]. In contrast, the lowest prevalence rates were observed in PAs of Dalesadi and Dalewabara districts where four species of *Glossina* were trapped. In 14.6 % of the PAs (*n* = 12), no trypanosomes were detected (Fig. 1). From our data, except for the higher susceptibility of the morsitans group to trypanosome infections, no clear explanation could be given for those variations (e.g. land cover, consistent link to a particular vector species). However, looking at the literature some common factors are associated with prevalence variability: annual and seasonal differences in sampling time, cattle abundance at watering places, micro-environmental ecological conditions [40–42], variations in tsetse and other hematophagous fly (mainly *Stomoxis* spp. and tabanids) densities and their respective vectorial capacity and seasonality [43–45] and the trypanotolerance/susceptibility of local cattle breeds [25, 40, 46].

The susceptibility of male cattle to trypanosome infections was higher than that of females. This can be explained by (i) their higher attractiveness for flies, (ii) a higher stress linked to their use as draft animals and (iii) the malnourishment of young males used prematurely for traction work with an insufficient food supply. Similar situations were reported in Ethiopia and Zambia [36, 47, 48].

#### Glossina

A total of 14,698 tsetse flies were trapped. Four different species were identified i.e. *G. tachinoides* (52 %), *G. pallidipes* (26 %), *G. morsitans submorsitans* (15 %) and *G. fuscipes fuscipes* (7 %). Significant differences in AT were observed between districts globally and for each of the *Glossina* species separately.

Qualitatively, Dale Sadi and Dale Wabera districts were colonized by four *Glossina* species whilst one, two or three species prevailed in the remaining 8 districts (Table 5). In Asosa district, only *G. m. submorsitans* was observed, which is probably due to the fact that Asosa is characterized by an unspoiled savannah environment, as this species is particularly sensitive to encroachment. Additionally this area is not drained by large rivers, which explains the absence of riverine flies. In the other districts, ecological features are more diverse, providing niches for different coexisting species. A number of reports have shown that the overlapping of two to three tsetse species is common [49, 50]. *G. pallidipes*, *G. m. submorsitans*, *G. tachinoides* and *G. f. fuscipes* were heterogeneously distributed in the western and north western zones of Ethiopia which is in agreement with previous studies [16, 17].

**Table 5:** The multivariable model presenting the risk factors associated with tsetse fly abundance in Ethiopia

Risk factor	Category	IRR	95 % CI	p-value
Altitude	≤1200 m^a^	1	-	-
	>1200 m, ≤1300 m	0.71	(0.67,0.74)	<0.001
	>1300 m, ≤1400 m	0.79	(0.76, 0.83)	<0.001
	>1400 m, ≤1500 m	0.34	(0.32,0.37)	<0.001
	>1500 m	0.70	(0.63,0.78)	<0.001

With *IRR* Incidence Rate Ratio, *CI* Confidence Interval, ^a^reference

Differences in the flies’ sex ratios were noticed for *G. m. submorsitans* (1 male to 1.37 females), *G. pallidipes* (1 male to 1.32 females) and *G. f. fuscipes* (1 male to 1.22 females). Females are over-represented by 22 to 37 % for each species. This is in agreement with the reports of Leak in 1999 [38] who reported that females would represent 70 % to 80 % in unbiased sampling. A number of causal factors for the sex ratio distortion in favour of females include (i) selective attractiveness of the trap [38, 51], (ii) a longer life expectancy of females compared to males [52], (iii) male reproducers transmitting X-bearing or a non-functional Y-bearing sperm [53] and (iv) a higher mortality rate of the males because of their higher susceptibility to insecticides [54].

Quantitatively, the AT varied from PA to PA. Indeed, different ecological contexts are obviously translated by different fly densities and species compositions [11]. Low altitude was significantly and positively associated with AT. Indeed, altitude directly influences ecological parameters such as vegetation cover and structure inducing specific microclimatic zones at different elevations [22, 38]. Additionally, lower temperature at night limits the pupal development and scarcer vegetation renders the environment more stressful for tsetse flies. In Nigeria an odds ratio of 0.91 per 50 m altitude increase was observed with an upper limit of 1800 m a.s.l. above which no tsetse flies were observed [55, 56]. Our data confirms this observation except for the sole PA of Abuna Gali were high catches were observed despite the altitude being above 1500 m. The data of this sole PA is pulling the entire category upwards. The most likely explanation for this, is a vegetation particularly dense for this altitude and the proximity of a permanent river that is connected by small seasonal tributaries to the river system of the Didessa wildlife reserve situated 80 km East from Abuna Gali.

Altitude, presence or absence of rivers, trees and wildlife are certainly determining factors for fly abundance but one of the main factors that remains is human encroachment. Indeed, tsetse and human population densities are negatively correlated and tsetse fail to survive in areas inhabited by more than 40 people per km^2^ [57]. This is particularly true for *G. m. submorsitans,* which is very sensitive to habitat degradation. The varying intensity of tsetse control in each region can somewhat contribute to the observed variations. Unfortunately, no data was available to perform the analysis to substantiate this possibility.

Despite many years spent on tsetse and trypanosomosis control, the problem persists due to mismanagement of available disease control means particularly in terms of geographical distribution and choice of control measures [2, 11].

### Relationship between tsetse abundance and trypanosome prevalence

No relationship between AT and trypanosomosis prevalence was found (Odds Ratio = 1, 95 % CI: [0.99; 1], *p* = 0.348) in the 30 PAs where the comparison was made. This is mainly due to the fact that at low densities the trypanosomosis prevalence varied a lot, from 0 to very high levels. This might be partially explained by an increased vectorial capacity of flies under adverse conditions [58]. Indeed, starvation and high ambient temperatures increase the susceptibility of tsetse flies to trypanosome infections [59, 60], hence increasing the rate of transmission with subsequently increased trypanosomosis prevalence in animals. Another factor that should not be immediately discarded is the possibility of mechanical transmission of trypanosomes by hematophagous flies as previously described for *T. vivax* and *T. congolense* [61–63] and more specifically in Ethiopia [64–67]. However, in our study, the tsetse abundance was not adjusted for the tsetse infection rate rendering the estimation of the real tsetse challenge impossible and consequently, the link between the tsetse challenge and AAT prevalence [68]. Further experimental data is thus needed to know if a reduction in tsetse challenge would lead to a significant reduction in trypanosomosis prevalence [40]. Furthermore, the link between AT and trypanosomosis prevalence is weakened by the fact that cattle bred in PAs located far from rivers and/or suitable tsetse habitats, travel longer distances to reach watering points and grasslands in the dry season: cattle is being brought to the vectors. During the rainy season, water and shade are widespread; cattle are walking shorter distances but flies are more dispersed in this tsetse-favouring environment. The vector host interface is thus facilitated.

## Conclusions

The present study provides a recent update on AT and on trypanosomosis prevalence in the southwest of Ethiopia together with the associated risk factors. Despite increasing human encroachment and vector/disease control operations tsetse are still abundant in the studied region and trypanosomosis remains an impediment to livestock health and production. The high trypanosomosis prevalence with low or nil ATs should be further investigated so that coordinated control strategies can be established.
